# An empirical study of diagnosis-related group classification for hospitalization costs in lumbar disk herniation patients treated in Chinese medicine hospitals in China

**DOI:** 10.3389/fpubh.2026.1882922

**Published:** 2026-09-10

**Authors:** Meng-en Chen, Jing-yu Yang, Jian-nan Cui, Xiao-xi Zhang, Zhi-wei Wang, Yan Wang, Hao-jia Hou, Qian Zhang

**Affiliations:** 1 School of Economics and Management, Anhui Agricultural University, Hefei, China; 2 School of Management, Beijing University of Chinese Medicine, Beijing, China; 3 School of Health Management, Gansu University of Chinese Medicine, Lanzhou, China; 4 School of Humanities, Beijing University of Chinese Medicine, Beijing, China; 5 Guang’anmen Hospital, China Academy of Chinese Medicine Sciences, Beijing, China; 6 Department of General Affairs, Sichuan Clinical Research Center for Cancer, Sichuan Cancer Hospital and Institute, Sichuan Cancer Center, University of Electronic Science and Technology of China, Chengdu, China

**Keywords:** Chinese medicine hospitals, DRG, hospitalization cost, LDH, payment system

## Abstract

**Objectives:**

Lumbar disk herniation (LDH) is a frequent and multiple disease that imposes a heavier global economic burden. As China’s diagnosis related group (DRG) payment system for Chinese medicine health insurance has entered the initial stage. Our study explored the factors influencing the hospitalization costs and established DRG cases classification scheme of LDH patients in Chinese medicine hospitals.

**Methods:**

A total of 2,857 LDH patients were hospitalized in Qingyang City Hospital of Chinese Medicine from January 2017 to June 2022. Using univariate analysis and multiple linear regression model to determine the primary factors influencing hospitalization costs for LDH patients. These factors were then utilized as classification node variables in a decision tree model to categorize LDH patients into diagnosis related groups (DRGs). The outcomes of these groupings were evaluated, and standard costs were computed for each DRG.

**Results:**

Hospitalization costs of patients with LDH were mainly affected by age, admission pathways, diagnosis and treatment based on Chinese medicine evidence, complications and comorbidities, surgery and operation, and length of stay, with the latter being the most crucial one. The decision tree model resulted in 10 DRGs with corresponding standard costs. The intra-group evaluation metric coefficient of variation (CV) was appropriate (required to be less than 1, actual range:0.23–0.91), and the inter-group evaluation metric reduction in variation (RIV) was suitable (needed to be greater than 0.4, actual value is 0.48). Significant variations exist in disease related weight (RW) and standard costs across all groups.

**Conclusion:**

The E-CHAID decision tree model grouping results are reasonably robust, showing good homogeneity within each DRG and notable heterogeneity between DRGs. Based on these groupings, scientifically formulate standard costs for each DRG and set upper limits, providing valuable insights to advance reforms in Chinese medicine DRG-based healthcare payment methods.

## 1 Introduction

The prevalence of back pain in society is notably high, with 70–85% of individuals experiencing it at some point in their lives (1). LDH is a common cause of back pain, neurological dysfunction, and pain in the buttocks or legs, with primarily results from degenerative changes in the lumbar disks, where partial or complete rupture of the annulus fibrosus (2, 3). Back pain has been recognized since ancient times, but its association with disk herniation was not identified until the early 20th century when Mixter and Barr first described it (4). LDH significantly impacts public health, potentially affecting 2–3% of the population. Among males aged 35 and older, the prevalence is 4.8%, while among females in the same age group, it is 2.5% (5). Furthermore, LDH remains a primary reason for spinal surgery in adults (5) and is a growing concern for global healthcare institutions, with modern sedentary work practices and an aging population (6).

In 2016, among the healthcare costs of 154 diseases in the United States, the expenditure for back and neck pain was high, estimated at $134.5 billion (7). From 2012 to 2016, the social cost of back pain in Brazil reached $2.2 billion, with productivity losses accounting for 79% of this cost, and total medical expenses estimated at $460 million (8). Research from Netherlands and Spain indicated that back pain burdens the national economy, with costs representing approximately 0.6% of their gross domestic products (9, 10). A study in the United Kingdom showed that total healthcare costs for patients with back pain were double those of a matched control group (£1,074 versus £516) (11). In 2021, the average hospitalization cost per admission for LDH patients was ¥26,730.2 in provincial hospitals and ¥41,136.2 in central hospitals in China, ranking among the highest across various disease categories at all hospital levels in the country (12). Overall, back pain imposes a substantial economic burden globally, with China experiencing an extremely affected population and a heavier disease burden.

Back pain is influenced by multiple factors (13), and LDH treatment falls into two main categories: surgical and non-surgical (5, 14, 15). Although surgical treatments such as high-intensity laser therapy can provide rapid symptom relief (16), the procedures themselves and the associated postoperative care carry higher risks and impose a substantial cost burden. Non-surgical treatments have been shown to offer effective symptom relief and facilitate recovery, often with a favorable cost-effectiveness profile (17–19). Traditional Chinese Medicine (TCM) predominantly employs non-surgical modalities, including acupuncture, massage therapy, traction, herbal medicine, and moxibustion. These interventions appear to alleviate symptoms and improve patients’ quality of life, potentially at a lower healthcare cost (20–23). In a related vein, Knezevic NN suggested that the prevention of back pain may represent a particularly effective approach to reducing healthcare expenditures, with traditional Chinese exercises such as Tai Chi and Five Animal Frolics being proposed as viable preventive options (24).

DRG is a classification system that categorizes cases based on the consistency of clinical processes and the similarity in resource consumption (20, 25). It has proven effective in cost control across various countries (26–29), serving as a tool for pricing, payment management, budget allocation, and hospital performance evaluation and comparison (30). In 2019, China’s National Healthcare Security Administration initiated nationwide DRG reform efforts to curb medical expenses and enhance healthcare services, achieving initial success in several comprehensive hospitals (31–34), with a recent emphasis on DRG reform and practices in TCM. Given the substantial global health burden of LDH and the considerable cost variations across different treatment approaches, it is important to explore the potential of TCM in alleviating the economic burden of this condition, particularly as LDH is recognized as a disease with distinctive TCM advantages and is supported by well-established clinical pathways and practice standards. Therefore, we conducted a study on the factors influencing inpatient costs and DRG case grouping payments for Chinese medicine treatment of LDH in Chinese medicine hospitals, with significant importance and value in advancing China’s healthcare security system and enhancing its healthcare insurance framework.

## 2 Materials and methods

### 2.1 Data sources

Through the Gansu Province Comprehensive Health Big Data Platform, we obtained the discharge summary information of patients from Qingyang City Hospital of Chinese Medicine (the earliest tertiary Chinese medicine hospital in Northwest China to implement DRG payment reform) from January 2017 to June 2022. Inclusion criteria: the primary ICD-10 diagnosis code is M51.202, and the TCM disease classification code is BNS150 (1995 edition) / A03.06.04.06.01 (2021 edition). Exclusion criteria: length of stay of less than 1 day or more than 90 days, zero hospitalization costs, and illogical medical record information that cannot be supplemented. A total of 2,857 records were collected. The valid data includes the patient’s gender, age, marital status, visit times, admission pathway, admission disease status, use of Chinese medicine diagnostic and therapeutic equipment, use of Chinese medicine diagnostic and treatment techniques, diagnosis and treatment based on Chinese medicine evidence, complications and comorbidities, surgeries and operations, length of stay, hospitalization costs, etc.

### 2.2 Statistical analysis

Before conducting statistical analysis, hospitalization costs for LDH patients at Qingyang City Hospital of Chinese Medicine from January 2017 to June 2022 were adjusted using the Gansu Province Healthcare Consumer Price Index (CPI) with 2016 as the base year, aiming to reduce potential research bias. The detailed CPI adjustment methodology is presented in Table 1.

**Table 1:** Consumer Price Index (CPI) for healthcare in Gansu Province (2017–2022).

Year	CPI	Cost adjustment method
2017	105.2%	Adjusted Cost_2017_ = Actual Cost_2017_/CPI_2017_
2018	108.0%	Adjusted Cost_2018_ = Actual Cost_2018_/CPI_2018_/CPI_2017_
2019	102.0%	Adjusted Cost_2019_ = Actual Cost_2019_/CPI_2019_/CPI_2018_/CPI_2017_
2020	100.6%	Adjusted Cost_2020_ = Actual Cost_2020_/CPI_2020_/CPI_2019_/CPI_2018_/CPI_2017_
2021	100.2%	Adjusted Cost_2021_ = Actual Cost_2021_/CPI_2021_/CPI_2020_/CPI_2019_/CPI_2018_/CPI_2017_
2022	100.5%	Adjusted Cost _2022_ = Actual Cost_2022_/CPI_2022_/CPI_2021_/CPI_2020_/CPI_2019_/CPI_2018_/CPI_2017_

The data analysis was performed using SPSS 26.0 software, with the details of the data coding and assignment processing of each variable shown in Table 2. Initially, the hospitalization costs of LDH patients were analyzed through univariate analysis. The Mann–Whitney U test was employed for dichotomous variables, while the Kruskal-Wallis H test was used for multichotomous variables, both presented as median and quartile. Secondly, variables that demonstrated statistical significance in the univariate analysis (*p* < 0.05) were used as independent variables to construct a multiple linear regression model, aiming to identify the primary factors impacting hospitalization costs. Subsequently, leveraging the findings from the multiple linear regression analysis of factors affecting hospitalization costs, categorical variables were identified to develop an E-CHAID decision tree model for categorizing LDH patients into DRGs. Finally, for the DRGs grouping results of LDH cases, evaluations were conducted using various methods and indicators, including non-parametric tests (Kruskal-Wallis H test), CV, RIV, and RW. A comprehensive analysis of the inpatient standard cost for each DRG group was then performed. The test level for the above statistical analysis was *α* = 0.05.

**Table 2:** Classification and assignment of variables.

Variable attributes	Variable codes	Variable names	Dummy variables	Variable assignment
Endogenous variables	*Y*	Hospitalization cost (CNY ¥)	—	Log (Hospitalization cost)
Exogenous variables	*X*_1_	Gender	—	0 = Male, 1 = Female
	*X*_2-0_ ~ *X*_2-2_	Age(years)	<45 (Reference)	0,0
			45 ~ 60	1,0
			>60	0,1
	*X*_3_	Marital status	—	0 = Married, 1 = Others
	*X*_4_	Visit times	—	0 = One time, 1 = Two or more times
	*X*_5_	Admission pathways	—	0 = Outpatient or emergency, 1 = Others
	*X*_6_	Admission disease status	—	0 = Determination, 1 = Undetermination or none
	*X*_7_	Use of Chinese medicine diagnostic and therapeutic equipment	—	0 = Yes,1 = No
	*X*_8_	Use of Chinese medicine diagnostic and treatment techniques	—	0 = Yes,1 = No
	*X*_9_	Diagnosis and treatment based on Chinese medicine evidence	—	0 = Yes,1 = No
	*X*_10_	Complications and comorbidities	—	0 = Yes,1 = No
	*X*_11_	Surgeries and operations	—	0 = Yes,1 = No
	*X*_12-0_ ~ *X*_12-3_	Length of stay (days)	1 ~ 7(Reference)	0,0,0
			8 ~ 14	1,0,0
			15 ~ 21	0,1,0
			22 and above	0,0,1

It is worth noting that the original hospitalization costs data does not follow a normal distribution, while the log-transformed hospitalization costs approximately do. To ensure rigorous and effective data analysis, Log (hospitalization costs) was used as the dependent variable in multiple linear regression and decision tree model. However, when estimating cost based on the results of influencing factors and the decision tree model, the original actual data could be used directly.

## 3 Results

### 3.1 Univariate analysis

**Figure 1:**
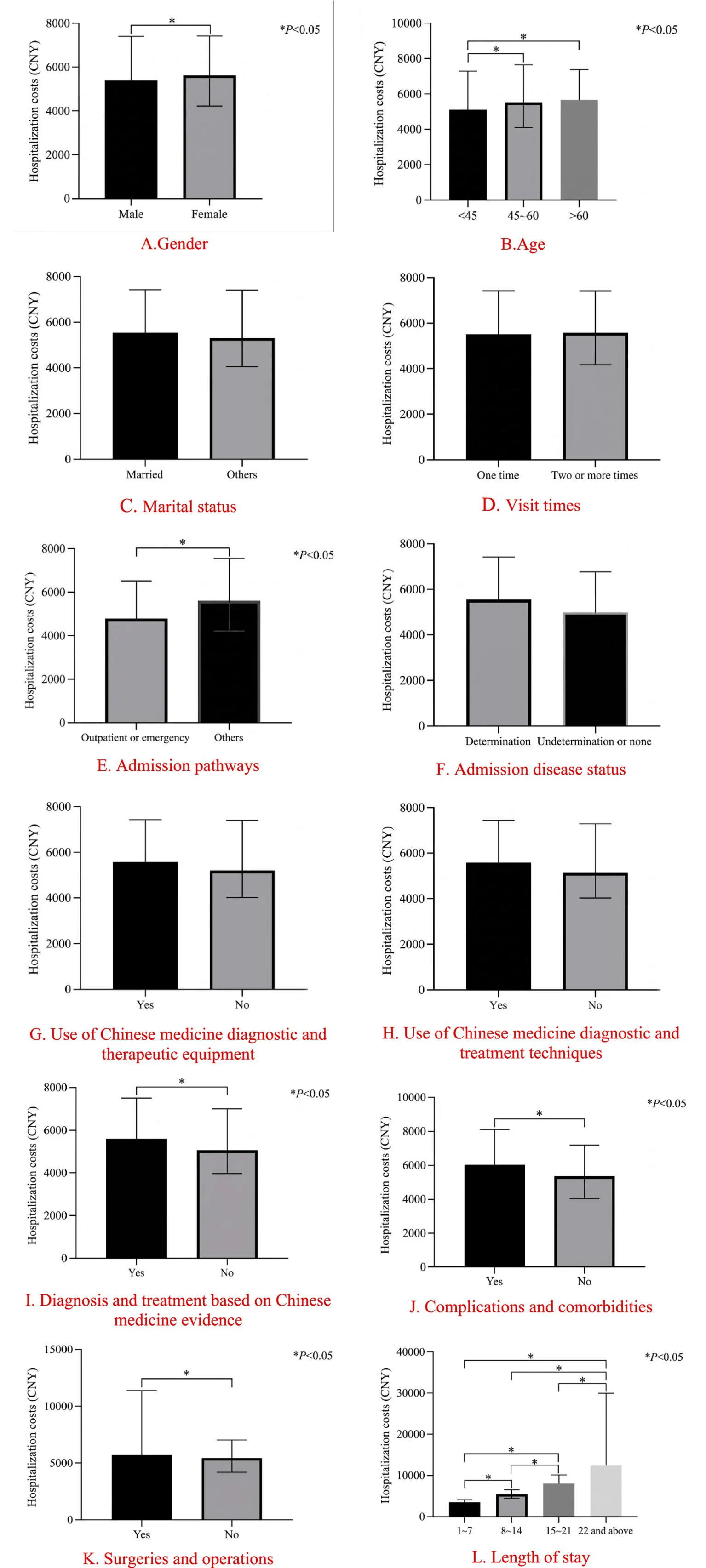
Comparison of hospitalization costs in LDH patients with different characteristics.

From the results of univariate analysis, we observed that hospitalization costs for patients with LDH, an advantageous disease in Chinese medicine, were associated with gender, age, admission pathway, diagnosis and treatment based on Chinese medicine evidence, complications and comorbidities, surgeries and operations, and length of stay (*p* < 0.05). Detailed findings are available in Table 3 and Figure 1.

**Table 3:** Description of univariate analysis of hospitalization costs.

Variables	Variable categories	*N* (%)	Hospitalization costs (CNY)/ *M* (*P*_25_, *P*_75_)	*Z/H*-value*^a^*	*p*-value
Gender	Male	1,235 (43.23)	5397.97 (4056.66, 7408.41)	−1.999	0.046
	Female	1,622 (56.77)	5617.17 (4232.49, 7426.15)		
Age(years)	<45	426 (14.91)	5097.60 (3659.64, 7287.09)	15.422	<0.001
	45 ~ 60	954 (33.39)	5517.85 (4107.29, 7644.75)		
	>60	1,477 (51.70)	5664.69 (4269.66, 7367.71)		
Marital status	Married	2,548 (89.18)	5531.09 (4167.72, 7425.91)	−0.862	0.389
	Others	309 (10.82)	5287.40 (4046.50, 7409.54)		
Visit times	One time	2,677 (93.70)	5506.58 (4158.56, 7425.91)	−0.467	0.641
	Two or more times	180 (6.30)	5578.05 (4174.50, 7419.50)		
Admission pathways	Outpatient or emergency	290 (10.15)	4783.23 (3919.03, 6513.00)	−4.430	<0.001
	Others	2,567 (89.85)	5602.65(4218.75, 7551.32)		
Admission disease status	Determination	2,791 (97.69)	5528.32 (4158.56, 7426.48)	−0.727	0.467
	Undetermination or none	66 (2.31)	4981.30 (4258.17, 6769.70)		
Use of Chinese medicine diagnostic and therapeutic equipment	Yes	2,293 (80.26)	5579.25 (4198.16, 7426.70)	−1.516	0.130
	No	564 (19.74)	5198.83(4018.62, 7401.38)		
Use of Chinese medicine diagnostic and treatment techniques	Yes	2,284 (79.94)	5592.24 (4196.56, 7443.79)	−1.831	0.067
	No	573(20.06)	5132.23 (4026.94, 7289.85)		
Diagnosis and treatment based on Chinese medicine evidence	Yes	2,421 (84.74)	5592.27 (4197.06, 7506.47)	−2.518	0.012
	No	436 (15.26)	5049.42(3963.67, 7003.57)		
Complications and comorbidities	Yes	734 (25.69)	6039.34 (4559.23, 8114.69)	−6.054	<0.001
	No	2,123 (74.31)	5353.20 (4038.74, 7201.91)		
Surgeries and operations	Yes	874 (30.59)	5730.84 (4049.87, 11378.66)	−5.294	<0.001
	No	1983 (69.41)	5457.32 (4184.07, 7043.91)		
Length of stay (days)	1 ~ 7	619 (21.67)	3483.04 (2858.67, 4121.69)	1600.276	<0.001
	8 ~ 14	1,505 (52.68)	5452.16 (4533.14, 6531.61)		
	15 ~ 21	503 (17.61)	8054.49 (6877.27, 10153.63)		
	22 and above	230 (8.05)	12385.42 (9197.59, 29942.08)		

M (P25, P75) median (the first quartile, the third quartile). ^a^Z/H-value: Mann–Whitney U test statistical value or Kruskal–Wallis H test statistical value.

### 3.2 Multiple linear regression

The statistically significant variables in the univariate analysis were incorporated into a multiple linear regression model, where the logarithm of hospitalization costs served as the dependent variable. The regression results revealed that age, admission pathway, diagnosis and treatment based on Chinese medicine evidence, complications and comorbidities, surgeries and operations, and length of stay are the main factors influencing hospitalization costs for LDH patients, with the regression equation of the patient’s hospitalization costs: *Y* = 3.480 + 0.054*X_2-1_ + 0.062*X_2-2_-0.058*X_5_-0.028*X_9_-0.031*X_10_ + 0.107*X_11_ + 0.225*X_12-1_ + 0.450*X_12-2_ + 0.660*X_12-3_ (*F* = 324.677, *p* < 0.001, *R*^2^ = 0.533). Collinearity statistics indicated that the variance inflation factor (VIF) values for each variable were below 5, indicating no issues with collinearity. The Durbin-Watson value was 1.804, close to 2, suggesting no autocorrelation issues in the model (Table 4). The regression results indicate that higher hospitalization costs are associated with LDH patients who are male, over 45 years old, admitted through outpatient or emergency, receiving diagnosis and treatment based on Chinese medicine evidence, presenting with complications and comorbidities, undergoing no surgeries and operations, and having a longer length of stay.

**Table 4:** Multiple linear regression results of hospitalization costs of LDH patients.

Variables	*B* ^b^	Std. Error	Beta^c^	*t*-value	*p*-value	Collinearity statistics	
						Tolerance	*VIF*
Constant	3.480	0.017		204.236	<0.001		
Gender (ref = Male)	−0.001	0.007	−0.002	−0.164	0.870	0.990	1.010
Age (ref = <45)							
Age(45 ~ 60)	0.054	0.011	0.095	5.016	<0.001	0.460	2.176
Age(>60)	0.062	0.010	0.116	6.133	<0.001	0.456	2.192
Admission pathways (ref = Outpatient or emergency)	−0.058	0.012	−0.065	−4.923	<0.001	0.935	1.070
Diagnosis and treatment based on Chinese medicine evidence (ref = Yes)	−0.028	0.010	−0.038	−2.918	0.004	0.959	1.042
Complications and comorbidities (ref = Yes)	−0.031	0.009	−0.050	−3.600	<0.001	0.850	1.176
Surgeries and operations (ref = Yes)	0.107	0.008	0.183	13.289	<0.001	0.861	1.161
Length of stay (ref = 1 ~ 7)							
Length of stay (8 ~ 14)	0.225	0.009	0.419	25.487	<0.001	0.607	1.647
Length of stay (15 ~ 21)	0.450	0.011	0.638	40.459	<0.001	0.660	1.515
Length of stay (22 and above)	0.660	0.014	0.669	46.189	<0.001	0.783	1.278
*R*^2^ -value	0.533						
*F* - value	324.677						
*p* - value	<0.001						
Durbin-Watson - value	1.804						

Age and Length of stay are ordinal category variables, whereas the regressions model only analyzed the factors associated with hospitalization costs, and we did not perform dummy variables regressions to fit the decision tree model. VIF variance inflation factor. ^b^B unstandardized coefficients, ^c^Beta standardized coefficients.

### 3.3 Decision tree model

Using the statistically significant variables from the multiple linear regression model as inputs, an E-CHAID decision tree model was constructed with the logarithm of hospitalization costs as the dependent variable to group DRG cases of LDH. The decision tree model was designed with a maximum depth of 3 layers, and the default minimum number of cases for parent and child nodes was set to 100 and 50, respectively. In addition, the E-CHAID decision tree model was validated using split-sample cross-validation. Cases were randomly assigned to the training and validation datasets, with 50% of the sample allocated to each dataset.

**Figure 2:**
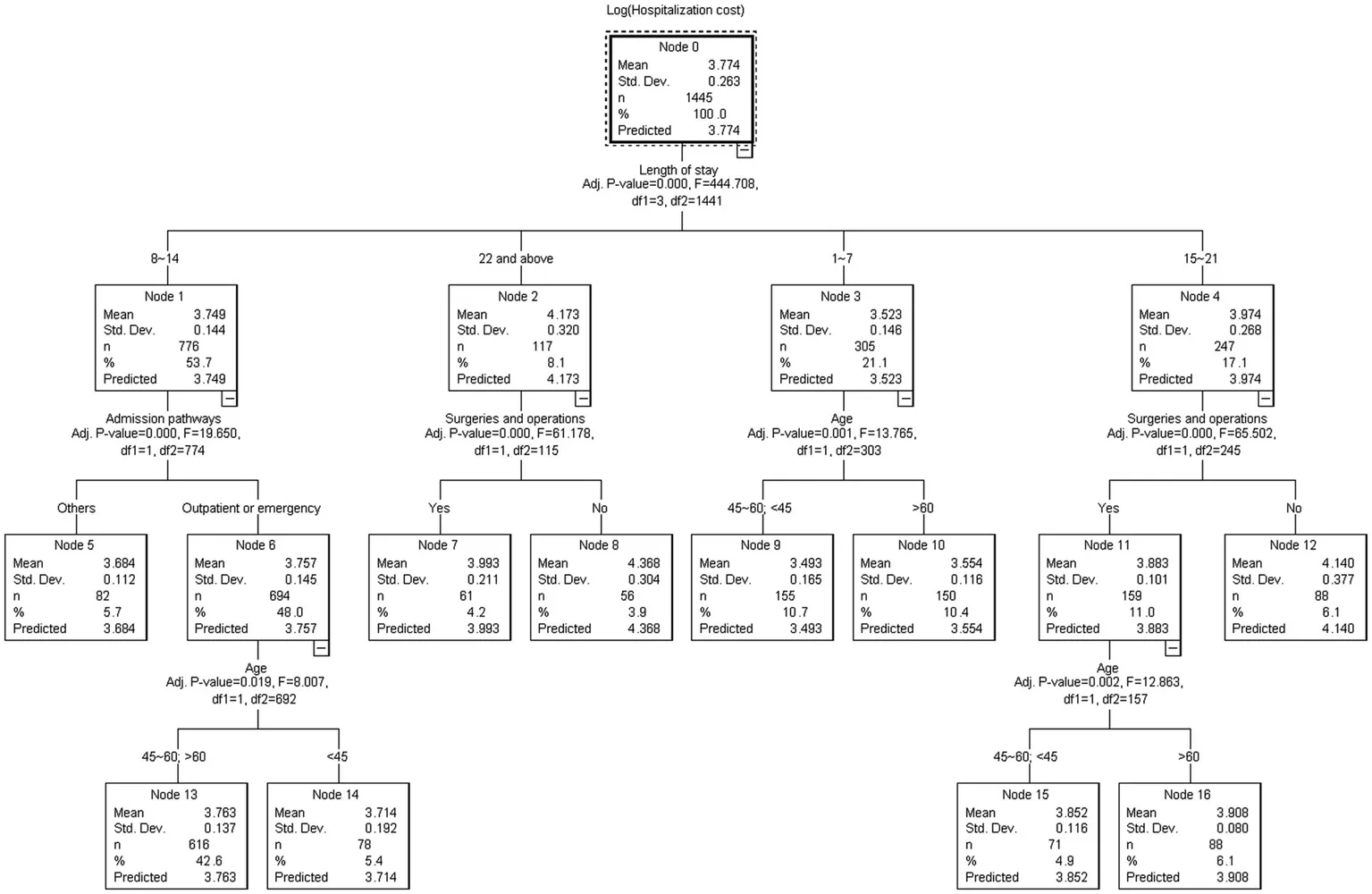
Training sample results of decision tree model for DRG grouping of LDH patients in Qingyang City Hospital of Chinese Medicine.

**Figure 3:**
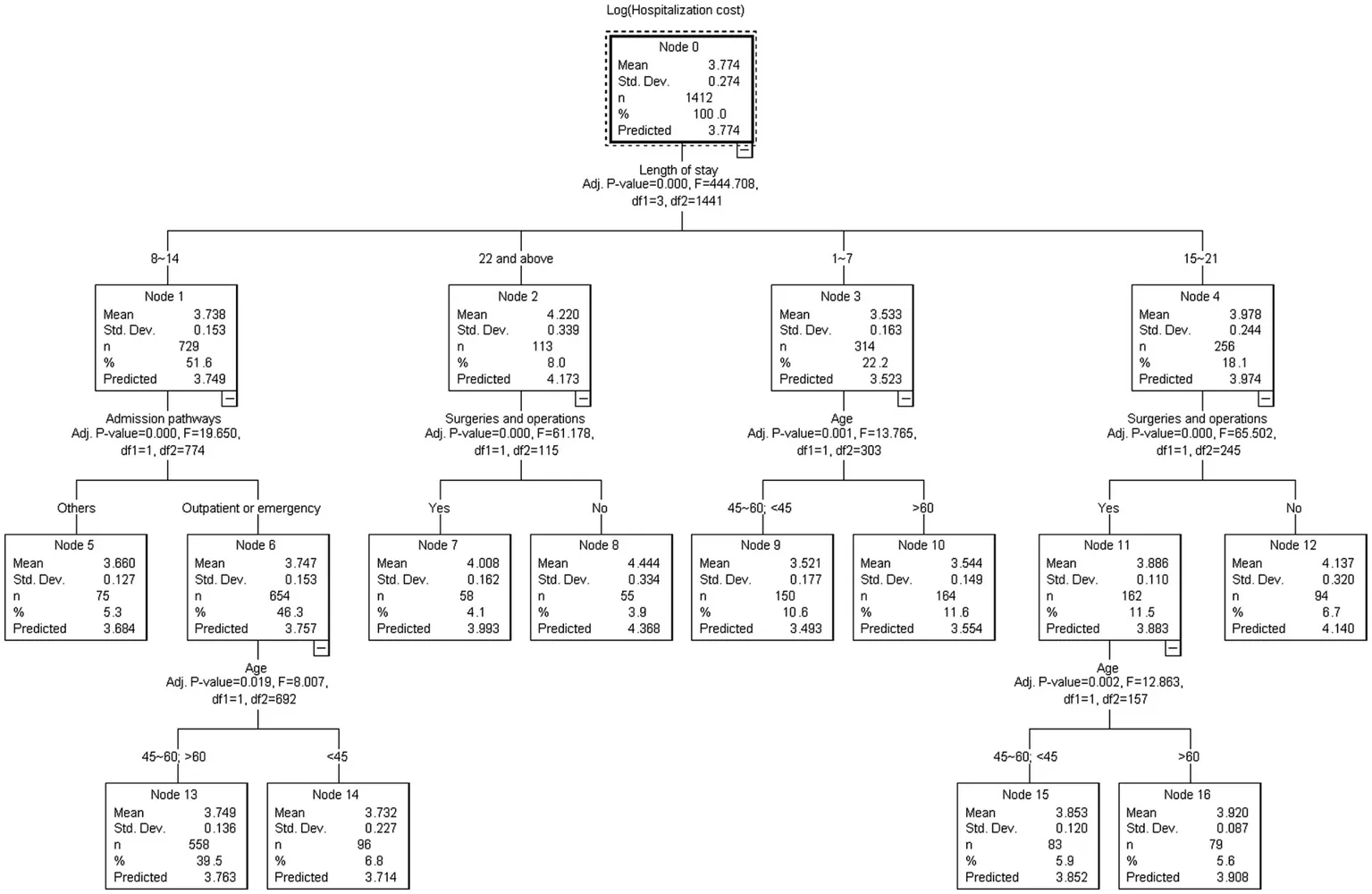
Test sample results of decision tree model for DRG grouping of LDH patients in Qingyang City Hospital of Chinese Medicine.

By developing a decision tree model, 2,857 cases of LDH were categorized into 10 DRGs. The first classification level was based on the length of stay, the second level considered the admission pathways, surgeries and operations, and age, with the third level accounting for age. The detailed classification process for the training and test sample of the decision tree model for LDH patients at Qingyang City Hospital of Chinese Medicine is illustrated in Figures 2, 3.

### 3.4 DRGs grouping evaluation

The cases of LDH at Qingyang City Hospital of Chinese Medicine were categorized into 10 DRGs, with specific details provided in Table 5. Among these groups, DRG-2 had the highest number of cases, accounting for 41.09%, while DRG-5 had the fewest cases at 4.17%. There was a notable disparity in case numbers across the various DRGs. Concerning median hospitalization costs, DRG-5 had the highest at CNY 28,835.47, whereas DRG-6 had the lowest at CNY 3,257.77, indicating significant cost variations between different DRGs. RW represents the ratio of each DRG’s average hospitalization costs to the overall average, reflecting their relative consumption of medical resources. DRG-5 had the highest related weight, and DRG-6 had the lowest. Detailed disease weights for each DRG can be found in Table 5.

**Table 5:** DRG grouped results and RW of LDH patients in Qingyang City Hospital of Chinese Medicine.

Groups	Case mix classification	*N*	Percentage (%)	Hospitalization costs (CNY)			CV	RW
				Mean	Median	Std. Dev.		
DRG-1	Length of stay (8–14 days), Admission pathways (Others)	157	5.50	4,878.28	4,598.58	1,300.13	0.27	0.63
DRG-2	Length of stay (8–14 days), Admission pathways (Outpatient or emergency), Age (≥45 years)	1,174	41.09	6,045.61	5,634.17	2,632.70	0.44	0.78
DRG-3	Length of stay (8–14 days), Admission pathways (Outpatient or emergency), Age (<45 years)	174	6.09	6,011.68	5,097.60	3,722.81	0.62	0.78
DRG-4	Length of stay (22 and above days), Surgeries and operations (Yes)	119	4.17	11,175.02	10,096.52	7,319.58	0.65	1.45
DRG-5	Length of stay (22 and above days), Surgeries and operations (No)	111	3.89	32,297.32	28,835.47	20,426.92	0.63	4.18
DRG-6	Length of stay (1–7 days), Age (≤60)	305	10.68	3,502.85	3,257.77	1,932.61	0.55	0.45
DRG-7	Length of stay (1–7 days), Age (>60)	314	10.99	3,731.15	3,649.22	1,822.84	0.49	0.48
DRG-8	Length of stay (15–21 days), Surgeries and operations (Yes), Age (≤60)	154	5.39	7,371.32	7,409.81	1,864.72	0.25	0.95
DRG-9	Length of stay (15–21 days), Surgeries and operations (Yes), Age (>60)	167	5.85	8,347.14	8,117.94	1,598.49	0.19	1.08
DRG-10	Length of stay (15–21 days), Surgeries and operations (No)	182	6.37	19,235.03	10,296.22	16,989.49	0.88	2.49

CV, Coefficient of variation; RIV, Reduction in variation.

The CV value is utilized to gage the uniformity of resource consumption within each DRG, with a smaller CV value indicating greater intra-group homogeneity (35). The RIV value assesses heterogeneity between groups, where a higher RIV value suggests increased inter-group diversity (36). In our study, the CV value for each DRG ranged from 0.19 to 0.88, indicating relatively consistent resource consumption within each group (CV < 1). The computed RIV value was 0.48, indicating notable inter-group heterogeneity (RIV > 0.40). Meanwhile, a non-parametric test (Kruskal-Wallis H test) was performed on hospitalization costs across the 10 DRGs, yielding statistically significant results (*H* = 1,666.331, *p* < 0.001). This underscores significant disparities in hospitalization costs among these 10 DRGs.

### 3.5 DRGs cost analysis

To mitigate the impact of extreme hospitalization costs on the study, the standard cost was derived using the arithmetic mean of the central 70% of hospitalization costs within each DRG (37). The upper-cost limit was established based on the 75th percentile of hospitalization costs within each DRG plus 1.5 times the interquartile range (38), as detailed in Table 6. Among these findings, DRG-3 had the highest proportion of cases exceeding the standard cost, with 16 cases surpassing the limit, accounting for 9.20%. Conversely, DRG-6 had the lowest proportion, with only 0 case (0.00%) exceeding the limit. Meanwhile, the standard cost is close to the median value of each DRG group. All DRGs had fewer than 10% of cases exceeding the standard cost, indicating a concentrated distribution of hospitalization costs within each group and minimal outliers. This suggested a reasonable grouping, indicating similar patterns of resource consumption within each DRG.

**Table 6:** Results of standard cost analysis for DRGs of LDH patients in Qingyang City Hospital of Chinese Medicine.

Groups	Case mix classification	Standard cost (CNY)	*P*_25_^d^ (CNY)	*P*_50_^e^ (CNY)	*P*_75_^f^ (CNY)	Interquartile distance (CNY)	Maximum cost (CNY)	Number of exceedances	Proportion of exceedance (%)
DRG-1	Length of stay (8–14 days), Admission pathways (Others)	4,783.41	4,059.00	4598.58	5,720.36	1,661.36	8,212.40	2	1.27
DRG-2	Length of stay (8–14 days), Admission pathways (Outpatient or emergency), Age (≥45 years)	5,677.02	4,711.23	5634.17	6,670.38	1,959.15	9,609.11	47	4.00
DRG-3	Length of stay (8–14 days), Admission pathways (Outpatient or emergency), Age (<45 years)	5,179.19	4,207.45	5097.60	6,211.55	2,004.10	9,217.71	16	9.20
DRG-4	Length of stay (22 and above days), Surgeries and operations (Yes)	10,154.37	7,881.74	10096.52	12,453.79	4,572.04	19,311.85	4	3.36
DRG-5	Length of stay (22 and above days), Surgeries and operations (No)	30,070.66	13,285.37	28835.47	47,879.28	34,593.91	99,770.15	0	0.00
DRG-6	Length of stay (1–7 days), Age (≤60)	3,306.06	2,647.17	3257.77	4,014.39	1,367.22	6,065.22	7	2.30
DRG-7	Length of stay (1–7 days), Age (>60)	3,636.57	3,060.78	3649.22	4,227.34	1,166.55	5,977.17	5	1.59
DRG-8	Length of stay (15–21 days), Surgeries and operations (Yes), Age (≤60)	7,333.03	6,054.15	7409.81	8,656.09	2,601.94	12,558.99	0	0.00
DRG-9	Length of stay (15–21 days), Surgeries and operations (Yes), Age (>60)	8,255.00	7,256.91	8117.94	9,386.41	2,129.50	12,580.66	2	1.20
DRG-10	Length of stay (15–21 days), Surgeries and operations (No)	14,793.64	7,239.18	10296.22	25,571.18	18,331.99	53,069.17	12	6.59

^d^P25 the first quartile, ^e^P50 the second quartile (median), ^f^P75 the third quartile.

## 4 Discussion

Our study is the first in China to conduct DRG grouping cost calculation and evaluation for patients with LDH, an advantageous disease in TCM hospitals, showcasing significant foresight and innovation. Through univariate and multiple analysis, we identified that hospitalization costs for patients with LDH were influenced by factors such as age, admission pathways, diagnosis and treatment based on Chinese medicine evidence, complications and comorbidities, surgeries and operations, and length of stay. In this study, hospitalization costs rose with patient age, echoing findings from various studies on healthcare expenditures (39–41). The trend primarily stems from older patients having poorer physical conditions, longer recovery periods, and higher medical service demands, resulting in escalated expenses.

Interestingly, admission pathways constitutes a meaningful predictor of hospitalization expenditure, with cost differentials primarily reflecting variations in patient severity across referral sources. Transfers from general to TCM hospitals—typically involving stable, post-acute cases—are associated with lower costs, whereas emergency- or outpatient-derived admissions, often characterized by greater clinical urgency, correlate with significantly elevated expenses. Besides, it was noteworthy that hospitalization costs for patients receiving diagnosis and treatment based on Chinese medicine evidence were higher than those for patients receiving non-differentiated care. This is because, after diagnosis and treatment based on Chinese medicine evidence, doctors integrate the patient’s physical condition and disease characteristics to design targeted diagnosis, treatment, and nursing plans with more detailed and comprehensive medical services, thus requiring higher expenses. What’s more, patients with complications and comorbidities have more complex conditions, necessitating greater medical support and resulting in higher medical expenses, as observed in studies by Pagano et al. (42) and Ahn et al. (43). While surgical interventions are conventionally presumed to be resource-intensive, our multivariate regression analysis revealed a counterintuitive association: patients who underwent surgeries or operations incurred significantly lower hospitalization costs, after adjusting for other covariates. This finding, which was consistent with the univariate analysis, also confirmed that advancing age, prolonged length of stay, and alternative admission pathways remained significant positive contributors to overall expenditure. Notably, the regression coefficients underscored that length of stay was the pivotal factor influencing hospitalization costs, consistent with findings from other disease research (44–47).

The decision tree model effectively grouped patients with LDH patients at Qingyang City Hospital of Chinese Medicine. Through the DRG grouping process, it became apparent that length of stay, admission pathways, surgeries and operations, and age were crucial determinants. Essentially, these four variables substantially influence hospitalization costs, leading patients admitted through outpatient or emergency, without surgeries and operations, or experiencing longer length of stay to be categorized into high-cost DRG groups. Furthermore, the decision tree model created 10 DRGs with significant inter-group cost disparities and high intra-group patient homogeneity, indicating effective grouping outcomes. This underscored the applicability of decision tree data mining techniques in case grouping within Chinese medicine hospitals, a consensus supported by studies such as those by Ma et al. (48), Zhi et al. (49), and Wu et al. (50). However, certain DRGs in the classification results showed disproportionately high costs, potentially linked to the distinctive treatment methods of Chinese medicine hospitals. Variations in individualized Chinese medicine treatments for the same condition can lead to notable cost differences within the same DRG, highlighting a critical area for attention in DRG payment reforms for Chinese medicine institutions. Having to agree with scientifically defining standard costs for each DRG and setting reasonable upper limits for LDH patients can support a monitoring system to promptly identify abnormal hospitalization costs. This proactive approach aids hospitals in adjusting treatment strategies promptly, ensuring patient health rights while minimizing unnecessary medical resource consumption and reducing the economic burden on patients due to illness. Of course, China’s DRG framework allows a certain proportion of outlier and special cases. Specifically, approximately 5% of high-cost cases are permitted to exceed the standard cost limit, and around 3% of complex cases qualify for fee-for-service reimbursement rather than fixed DRG-based payment. These mechanisms help balance cost containment with the need to ensure appropriate patient quality of care and protect hospitals’ financial sustainability. In addition, a multi-tiered supervision system—including internal DRG management platforms, unannounced inspections by healthcare insurance authorities, and patient feedback mechanisms—collectively incentivize hospitals to control costs without compromising treatment quality. As China’s healthcare security system continues to develop, we expect further refinements in safeguarding patient rights and protecting the legitimate interests of medical institutions.

To control hospitalization costs for LDH patients in TCM hospitals, the main priority is to reduce length of stay while maintaining treatment efficacy. This approach can significantly lower medical resource consumption. Length of stay currently serves as a useful interim measure, but future indicators should give more weight to the value and effectiveness of therapeutic outcomes. Following this, Chinese medicine hospitals should actively employ their distinctive Chinese medicine rehabilitation techniques like tuina, massage, acupuncture, and other non-surgical therapies to aid patients with LDH. Moreover, the unique Chinese medicine diagnostic and therapeutic systems, encompassing Chinese medicine differential nursing, techniques, and equipment usage, play a crucial role in influencing treatment outcomes and hospitalization costs. Given the advantage disease of Chinese medicine such as LDH, Chinese medicine hospitals should capitalize on their specialized treatment methods to promote patient health while alleviating economic burdens.

Particularly amidst the current DRG medical insurance payment reforms in China’s Chinese medicine hospital system, government authorities and hospitals themselves need to prioritize the unique diagnostic and therapeutic systems of Chinese medicine. This entails timely updates and enhancements to Chinese medicine medical record schemes, improvements in Chinese medicine information technology infrastructure, and the development of DRG case grouping systems and models that reflect the distinctive features of Chinese medicine. However, in implementing DRG payment reforms for LDH, particular attention must be paid to potential variations in disease severity, socioeconomic status among patients, and heterogeneity in healthcare providers. First, a precise assessment system for LDH severity should be established to guide differentiated clinical treatment plans and corresponding DRG payment standards. Second, the impact of patients’ socioeconomic status on their financial burden should be taken into account; DRG-based medical insurance payment policies should incorporate policy tilting mechanisms for low-income populations to alleviate their out-of-pocket expenses. Furthermore, differentiated payment coefficients should be introduced to reflect the qualifications and service levels of healthcare providers. For instance, higher reimbursement coefficients could be allocated to senior experts to acknowledge their professional value and incentivize high-quality care delivery. All efforts are essential to effectively enhance public health with Chinese medicine and reduce the economic burden of diseases.

## 5 Limitations

Several limitations should be acknowledged. Our data came from a single institution, Qingyang City Hospital of Chinese Medicine, which limits generalizability. This hospital was chosen because it is one of the few tertiary TCM facilities in China that has implemented DRG payment reforms, making it a valuable starting point despite the single-site constraint. Our grouping analysis was confined to the TCM system without a Western medicine comparator, as comparable data from Western medicine hospitals were not accessible. Future studies could address this gap by drawing on Western medicine methodologies to refine TCM grouping strategies. In addition, our cost-only analysis did not include efficacy measures such as pain scores, functional recovery, or readmission rates, nor did we adjust for disease severity, detailed TCM technique categories, or socioeconomic status. These variables were not available in our homepage system data. These omissions may introduce bias and should be addressed in future research with richer clinical data.

## 6 Conclusion

Hospitalization costs for LDH patients in Chinese medicine hospitals are primarily influenced by age, surgeries and operations, admission pathways, complications and comorbidities, diagnosis and treatment based on Chinese medicine evidence, and length of stay, with the latter being the most crucial one. The decision tree model demonstrates the effective grouping of DRG cases for LDH patients in Chinese medicine hospitals, providing valuable insights for medical insurance payment reforms aimed at enhancing the treatment of LDH and other advantageous diseases of Chinese medicine.

## Data Availability

The original contributions presented in the study are included in the article/supplementary material, further inquiries can be directed to the corresponding author/s.
